# Sorcin couples Annexin A11 recruitment and ESCRT-III assembly during plasma membrane repair

**DOI:** 10.64898/2026.04.10.717788

**Published:** 2026-04-13

**Authors:** Jordan Matthew Ngo, Justin Krish Williams, Abinayaa Murugupandiyan, Randy Schekman

**Affiliations:** 1 Department of Molecular and Cell Biology, University of California, Berkeley, CA 94720; 2 Current Address: Cell Biology Program, Sloan Kettering Institute, New York, NY 10065; 3 Howard Hughes Medical Institute, University of California Berkeley, CA 94720

## Abstract

The absence of a cell wall affords animal cells diverse functionality at the cost of acute sensitization to plasma membrane (PM) damage. Thus, animal cells tightly monitor and maintain the integrity of their PM to prevent cell death. Genetic loss of PM repair factors is associated with human diseases including muscular dystrophy and neurodegeneration. Despite evidence that annexin and endosomal sorting complex required for transport (ESCRT) proteins are required for PM repair, the extent to which their recruitment is coordinated at sites of membrane damage is unclear. Here, we identify sorcin as a new PM repair factor that directly couples annexin A11 (ANXA11)-mediated sensing of PM damage and ESCRT-III assembly. We demonstrate that ANXA11, recruited to the PM upon damage-induced calcium influx, serves as an anchor that facilitates the sequential recruitment of sorcin and ESCRT-III at PM lesions. Our data highlight mechanistic and topological similarities between the budding of membrane-enveloped viruses and damage-induced microvesicles. We propose that they share a common mechanism of membrane budding and speculate that membrane-enveloped viruses may have co-opted this host pathway of PM ESCRT recruitment to facilitate virion assembly and propagation.

## Introduction

A defining feature of animal cells is the lack of a cell wall. Although this absence confers animal cells with distinct functional advantages compared to cells from other taxa, it also renders animal cells susceptible to plasma membrane (PM) damage. PM disruption is common, and a subset of cells, particularly those found in mechanically active tissues, are especially vulnerable to damage (McNeil and Steinhardt, 2003). For example, muscle and endothelial cells experience high levels of PM damage due to repeated contractions and fluid shear stress, respectively (Andrews and Corrotte, 2018; Horn and Jaiswal, 2018; Dias and Nylandsted, 2021). If unresolved, persistent PM damage leads to cell death. Accordingly, human cells tightly monitor and maintain PM integrity.

Extracellular calcium is essential for PM repair (Terasaki et al., 1997). Upon PM damage, calcium flows from the extracellular space into the cytoplasm and initiates the recruitment of membrane repair factors to the lesion site (McNeil and Steinhardt, 2003). Annexins are cytosolic proteins that bind phospholipids in the presence of calcium (Gerke and Moss, 2002) and participate in various aspects of PM repair including membrane patching and tension reduction (McNeil and Steinhardt, 2003; Gerke et al., 2024). Consistent with an important role in PM repair, genetic depletion of annexins in cultured cells impairs membrane resealing (McNeil et al., 2006; Bouter et al., 2011; Demonbreun et al., 2016; Koerdt and Gerke, 2017; Foltz et al., 2021), and loss of annexin expression or function in mice causes muscular dystrophy (Swaggart et al., 2014; Defour et al., 2017; Croissant et al., 2021).

In addition to annexins, the endosomal sorting complex required for transport (ESCRT) machinery promotes PM repair (Jimenez et al., 2014). The core ESCRT machinery, consisting of ESCRT-0, ESCRT-I, ESCRT-II, ESCRT-III, ALIX and Vps4, were originally identified for their role in sorting ubiquitylated membrane proteins into the intraluminal vesicles of multivesicular bodies (Katzmann et al., 2001; Schöneberg et al., 2017). However, it is now clear that the ESCRT machinery mediates reverse-topology membrane scission in diverse cellular processes including nuclear envelope sealing, autophagosome closure, cytokinesis, viral budding and lysosome repair (Vietri et al., 2020; Zhen et al., 2021; Burigotto and Carlton, 2025). Interestingly, ESCRT I-III are recruited to damaged lysosomes (Skowyra et al., 2018; Goul et al., 2026), whereas only ESCRT-III is recruited to PM lesions (Scheffer et al., 2014). Upon PM disruption, annexin A7 (ANXA7) localizes to the membrane lesion and recruits ALG-2, which subsequently recruits ESCRT-III (Sønder et al., 2019). However, recent studies have suggested that ALG-2 can directly bind membranes in the presence of calcium (Shukla et al., 2022; Shukla et al., 2024). Thus, the extent to which annexin recruitment is coupled to ESCRT-III assembly during PM repair is unclear.

Mutations in annexin A11 (ANXA11) and CHMP2B (a member of ESCRT-III) have been linked to amyotrophic lateral sclerosis (ALS), frontotemporal dementia (FTD) and limb-girdle muscular dystrophy (Skibinski et al., 2005; Smith et al., 2017; Johari et al., 2022; Martins et al., 2025), and a recent report has indicated that these mutations inhibit PM repair (Heffner et al., 2024). Given increasing evidence that PM repair is compromised in neurodegeneration and certain types of muscular dystrophy, we sought to identify novel factors required for this essential membrane repair pathway.

## Results

### Identification of proteins recruited to the inner leaflet of the PM upon calcium influx

**Figure 1.**
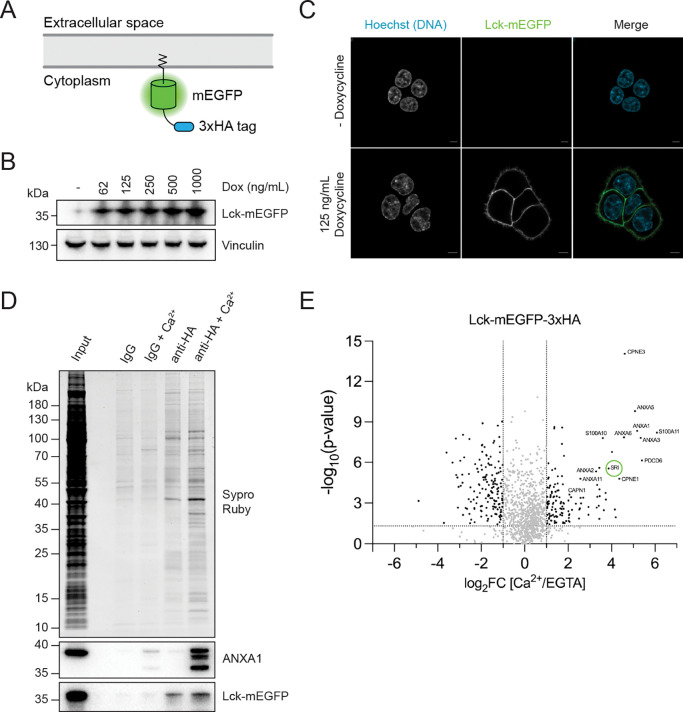
Quantitative organellar proteomics identifies calcium-dependent plasma membrane-binding proteins. (A) Schematic illustrating the membrane topology of Lck-mEGFP-3xHA when inserted into the plasma membrane. (B) Immunoblot analysis of HCT116 Lck-mEGFP-3xHA cells treated with increasing concentrations of doxycycline for 24 h. (C) Airyscan microscopy of HCT116 Lck-mEGFP-3xHA cells treated with vehicle (H_2_O) or 125 ng/ml doxycycline for 24 h. Cyan: DNA (Hoechst 33342); Green: Lck-mEGFP-3xHA. Scale bar: 5 μm. (D) Total protein (Sypro Ruby staining) analysis of post-nuclear supernatant input, IgG and anti-HA immunoprecipitants is shown. (E) Volcano plot showing proteins enriched in the anti-HA immunoprecipitants in the presence of calcium. Sorcin (SRI) is circled in green. Statistical significance was calculated using an unpaired two-tailed Student’s *t*-test.

Calcium influx upon PM disruption recruits a repertoire of membrane repair proteins to the lesion site (Andrews and Corrotte, 2018). We sought to leverage this feature of PM repair to identify new repair factors. We designed a membrane fractionation strategy that would allow us to identify proteins that are recruited to the surface of inside-out PM vesicles in the presence of calcium, reasoning that some of these factors might promote PM repair. We generated an HCT116 cell line that inducibly expressed monomeric enhanced green fluorescent protein (mEGFP) fused to the PM targeting sequence of Lck and a 3xHA epitope tag (Lck-mEGFP-3xHA) (Fig. 1A). Incubation of these cells with doxycycline induced titratable expression of Lck-mEGFP-3xHA (Fig. 1B), and visualization of these cells after doxycycline induction revealed specific labeling of the PM with mEGFP (Fig. 1C).

To identify proteins that were recruited to the PM inner leaflet upon calcium influx, we induced Lck-mEGFP-3xHA expression, mechanically ruptured cells and performed IgG or anti-HA immunoprecipitations in the absence or presence of calcium. We observed the recruitment of multiple proteins to the surface of inside-out PM vesicles upon addition of calcium (Fig. 1D). Annexin A1 (ANXA1) served as a positive control and was greatly enriched in the anti-HA + calcium immunoprecipitation. Label-free quantitative mass spectrometry analysis of the anti-HA immunoprecipitants identified many proteins that were reproducibly recruited to the inner leaflet of the PM in the presence of calcium (Fig. 1E).

Many of the significant hits identified in our mass spectrometry analysis have been previously implicated in PM repair. For example, we identified multiple members of the annexin family, S100A10/11 and ALG-2. We also identified calpains, which we recently demonstrated cleave annexins for PM repair (Williams et al., 2025). Of particular interest, we found the protein sorcin (SRI) as a significant hit in our analysis. Although sorcin has not been previously implicated in PM repair, it emerged as a top candidate gene in a genome-wide CRISPR interference screen that we performed to identify factors whose depletion sensitized cells to the pore-forming toxin perfringolysin O (PFO) in the presence of extracellular calcium (Williams and Ngo et al., 2026).

### Sorcin is recruited to PM lesions upon calcium influx and required for membrane repair

**Figure 2.**
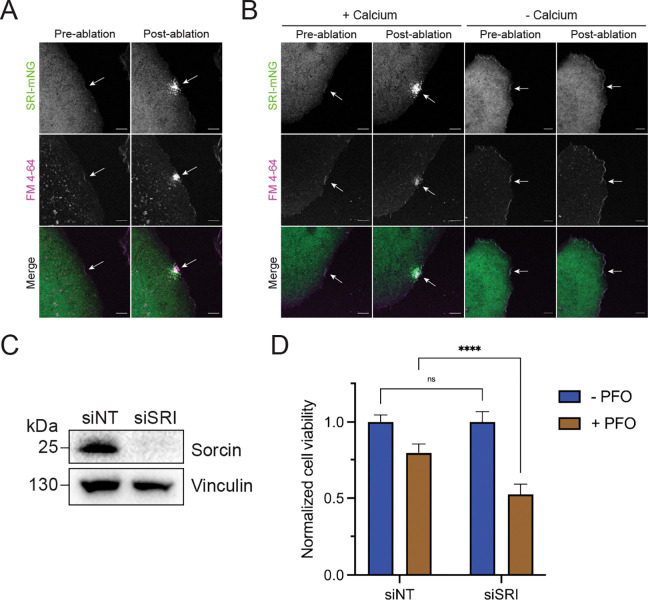
Sorcin is recruited to PM lesions upon calcium influx and required for membrane repair. (A) Representative confocal micrographs of U2-OS Sorcin-mNeonGreen (SRI-mNG) cells subjected to laser ablation wounding. Images shown are pre- and post-laser ablation. Green: SRI-mNG; Magenta: FM4–64. Scale bar: 5 μm. See corresponding Movie 1. (B) Representative confocal micrographs of U2-OS SRI-mNG cells subjected to laser ablation wounding in the presence or absence of extracellular calcium. Images shown are pre- and post-laser ablation. Green: SRI-mNG; Magenta: FM4–64. Scale bar: 5 μm. See corresponding Movies 2 and 3. (C) Immunoblot analysis of U2-OS wild-type cells transfected with control (siNT) or sorcin-targeting (siSRI) siRNAs. (D) Normalized cell viability of U2-OS siNT and siSRI cells following treatment with vehicle or perfringolysin O (PFO; 25 ng/ml). Graph displays mean ± SD (n = 9). Statistical significance was calculated using a two-way ANOVA (ns = not significant, ****p<0.0001).

After identifying sorcin as a potential PM repair factor, we tested whether sorcin is recruited to PM lesions. We generated a U2-OS cell line expressing fluorescently tagged sorcin (SRI-mNG) to monitor sorcin dynamics after laser ablation wounding. FM4–64 is a membrane impermeable dye that brightly labels the site of damage and the PM repair cap. Upon laser ablation, we observed FM4–64 staining and sorcin recruitment at the site of PM damage (Fig. 2A; Movie 1). Because extracellular calcium is essential for PM repair (Terasaki et al., 1997), we wondered whether calcium influx was required for the recruitment of sorcin to PM lesions. Laser ablation experiments revealed that the removal of extracellular calcium completely blocked both PM repair cap formation and sorcin recruitment to PM lesions (Fig. 2B; Movies 2–3).

We then asked whether sorcin depletion sensitized cells to PM damage. To address this, we depleted sorcin by RNA interference (Fig. 2C) and treated control (siNT) and sorcin knockdown (siSRI) cells with PFO (Fig. 2D). We observed that sorcin depletion sensitized cells to PFO treatment. These experiments established sorcin as a bona fide PM repair factor.

### Sorcin simultaneously binds ANXA11 and ALIX in the presence of calcium

Prompted by our observation that calcium influx is essential for the recruitment of sorcin to PM lesions (Fig. 2B), we sought to identify proteins that interact with sorcin in the presence of calcium. Sorcin, like ALG-2, is a member of the penta-EF-hand (PEF) family of proteins (Maki et al., 2002). Sorcin was previously reported to interact with a recombinant N-terminal fragment of annexin A11 (ANXA11) (Brownawell and Creutz, 1997), the strongest calcium-dependent candidate gene identified in our genetic screen for cell survival upon PFO challenge (Williams and Ngo et al., 2026). Additionally, ALG-2 has been demonstrated to bind ALIX (Missotten et al., 1999). Motivated by these results, we hypothesized that sorcin associates with ANXA11 and components of the ESCRT-III complex in the presence of calcium.

**Figure 3.**
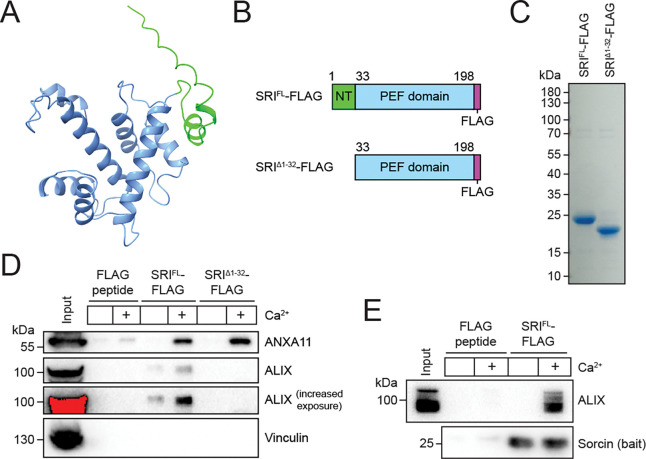
Sorcin simultaneously binds ANXA11 and ALIX in the presence of calcium. (A) Alphafold3 predicted protein structure of human sorcin (SRI). The flexible N-terminal domain (NT) is colored green, and the penta-EF hand (PEF) domain is colored blue. (B) Domain architecture of full-length (SRI^FL^-FLAG) and truncated (SRI^Δ1–32^-FLAG) sorcin constructs. (C) Coomassie-stained SDS-PAGE gel showing the purification of SRI^FL^-FLAG and SRI^Δ1–32^-FLAG. (D) Immunoblot analysis of cytosol input and FLAG peptide, SRI^FL^-FLAG and SRI^Δ1–32^-FLAG immunoprecipitants in the absence or presence of calcium. (E) Immunoblot analysis of recombinant protein input and immunoprecipitants from an SRI^FL^-FLAG/ALIX *in vitro* binding assay.

Alphafold3 modeling revealed that sorcin consists of two domains: a flexible N-terminal domain (amino acids 1–32) and a PEF domain (amino acids 33–198) (Fig. 3A). Given previous evidence indicating that the N-terminus of PEF proteins regulates their association with other proteins (Maki et al., 2012), we purified FLAG-tagged full-length (SRI^FL^-FLAG) and truncated (SRI^Δ1–32^-FLAG) sorcin from bacterial cells (Fig. 3B–C) for immunoprecipitation experiments. Full-length and truncated sorcin were immobilized on anti-FLAG beads, incubated with cytosol in the absence or presence of calcium, and analyzed by immunoblotting (Fig. 3D). These experiments revealed that sorcin bound to ANXA11 and ALIX in the presence of calcium. Notably, truncated sorcin retained its ability to bind ANXA11, but not ALIX. We then asked whether sorcin directly associates with ALIX. Immunoblot analysis of *in vitro* binding assays with recombinant proteins revealed that sorcin directly binds ALIX in the presence of calcium (Fig. 3E). Together, these data indicated that sorcin simultaneously binds ANXA11 and ALIX in the presence of calcium.

### ANXA11 is required for membrane repair and sorcin recruitment to PM lesions

**Figure 4.**
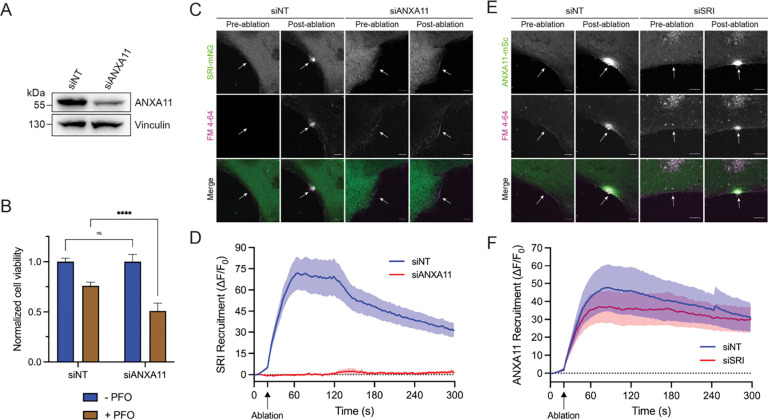
ANXA11 is required for PM repair and sorcin recruitment to PM lesions. (A) Immunoblot analysis of U2-OS wild-type cells transfected with control (siNT) or ANXA11-targeting (siANXA11) siRNAs. (B) Normalized cell viability of U2-OS siNT and siANXA11 cells following treatment with vehicle or perfringolysin O (PFO; 25 ng/ml). Graph displays mean ± SD (n = 6). Statistical significance was calculated using a two-way ANOVA (ns = not significant, ****p<0.0001). (C) Representative confocal micrographs of U2-OS Sorcin-mNeonGreen (SRI-mNG) siNT and siANXA11 cells subjected to laser ablation wounding. Images shown are pre- and post-laser ablation. Green: SRI-mNG; Magenta: FM4–64. Scale bar: 5 μm. See corresponding Movies 4 and 5. (D) Quantification of the recruitment dynamics of SRI-mNG cells transfected with siNT (n = 19) or siANXA11 (n = 14) siRNAs. Graph displays mean ± S.E.M. (E) Representative confocal micrographs of U2-OS ANXA11-mScarlet (ANXA11-mSc) siNT and sorcin knockdown (siSRI) cells subjected to laser ablation wounding. Images shown are pre- and post-laser ablation. Green: ANXA11-mSc; Magenta: FM4–64. Scale bar: 5 μm. See corresponding Movies 6 and 7. (F) Quantification of the recruitment dynamics of ANXA11-mSc cells transfected with siNT (n = 9) or siSRI (n = 13) siRNAs. Graph displays mean ± S.E.M.

Our sorcin truncation data led us to hypothesize that sorcin may couple ANXA11 recruitment and ESCRT-III assembly during PM repair. We thus evaluated the role of ANXA11 in PM repair and sorcin recruitment to PM lesions. We first tested whether ANXA11 was required for PM repair. We depleted ANXA11 using RNA interference (Fig. 4A) and treated siNT and ANXA11 knockdown (siANXA11) cells with PFO (Fig. 4B). This experiment revealed that ANXA11 depletion sensitized cells to PFO. We then asked whether ANXA11 is required for sorcin recruitment to PM lesions. Laser ablation studies revealed that ANXA11 depletion blocked the recruitment of sorcin to PM lesions (Fig. 4C–D; Movies 4–5). In contrast, sorcin depletion did not affect the recruitment of ANXA11 to PM lesions (Fig. 4E–F; Movies 6–7). Additionally, we noted that PM repair cap formation (as indicated by FM4–64 staining) was defective in ANXA11-depleted cells (Fig. 4C).

### Sorcin and ANXA11 are required for ESCRT-III recruitment to PM lesions

**Figure 5.**
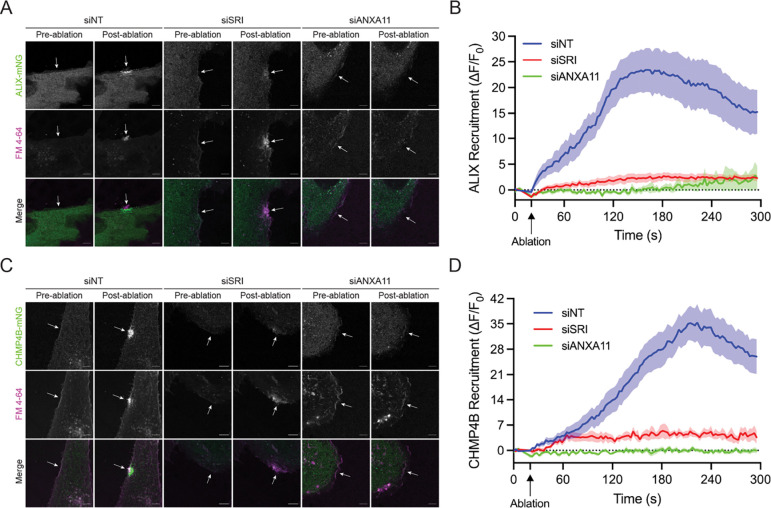
Sorcin and ANXA11 are required for ESCRT-III recruitment to PM lesions. (A) Representative confocal micrographs of U2-OS ALIX-mNeonGreen (ALIX-mNG) control (siNT), sorcin knockdown (siSRI) and ANXA11 knockdown (siANXA11) cells subjected to laser ablation wounding. Images shown are pre- and post-laser ablation. Green: ALIX-mNG; Magenta: FM4–64. Scale bar: 5 μm. See corresponding Movies 8–10. (B) Quantification of the recruitment dynamics of ALIX-mNG cells transfected with siNT (n = 15), siSRI (n = 12) or siANXA11 (n = 11) siRNAs. Graph displays mean ± S.E.M. (C) Representative confocal micrographs of U2-OS CHMP4B-mNeonGreen (CHMP4B-mNG) siNT, siSRI and siANXA11 cells subjected to laser ablation wounding. Images shown are pre- and post-laser ablation. Green: CHMP4B-mNG; Magenta: FM4–64. Scale bar: 5 μm. See corresponding Movies 11–13. (D) Quantification of the recruitment dynamics of CHMP4B-mNG cells transfected with siNT (n = 15), siSRI (n = 7) or siANXA11 (n = 7) siRNAs. Graph displays mean ± S.E.M.

After establishing a role for ANXA11 in PM repair and sorcin recruitment to PM lesions, we asked whether sorcin and ANXA11 were required for ESCRT-III recruitment. To address this, we depleted either sorcin or ANXA11 in U2-OS cells expressing fluorescent ALIX and CHMP4B fusion proteins for laser ablation studies. Strikingly, depletion of sorcin or ANXA11 blocked the recruitment of ALIX (Fig. 5A–B; Movies 8–10) and CHMP4B (Fig. 5C–D; Movies 11–13) to PM lesions.

### Sorcin is recruited to PM lesions together with ANXA11 and before ESCRT-III

**Figure 6.**
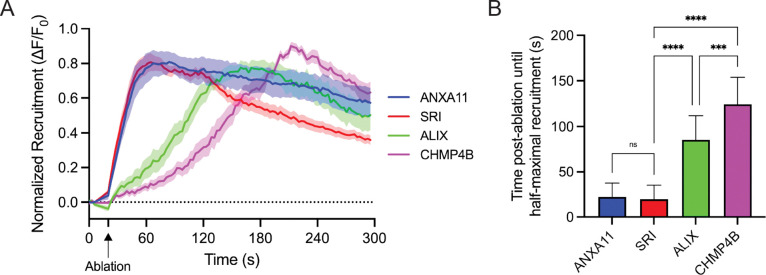
Sorcin is recruited to PM lesions together with ANXA11 and before ESCRT-III. (A) Quantification of the recruitment dynamics of ANXA11-mSc (n = 9), Sorcin-mNG (n = 19), ALIX-mNG (n = 15) and CHMP4B-mNG (n = 15) cells transfected with a non-targeting siRNA (siNT) from Figures 4D, 4F, 5B and 5D. Graph displays mean ± S.E.M. (B) Quantification of the time post-ablation until half-maximal recruitment of ANXA11-mSc (n = 9), Sorcin-mNG (n = 19), ALIX-mNG (n = 15) and CHMP4B-mNG (n = 15) siNT cells from Figures 4D, 4F, 5B and 5D. Graph displays mean ± SD. Statistical significance was calculated using a one-way ANOVA (ns = not significant, ***p<0.001, ****p<0.0001).

We then sought to establish the chronology in which ANXA11, sorcin, ALIX and CHMP4B are recruited to sites of PM damage. We re-analyzed our laser ablation data (Fig. 4–5) and quantified the kinetics by which each protein is recruited to PM lesions (Fig. 6A–B). This analysis revealed that sorcin and ANXA11 are simultaneously recruited to PM lesions, followed by ALIX then CHMP4B. These data, taken together with our truncation (Fig. 3) and laser ablation (Fig. 4–5) data, suggested a model in which ANXA11 at PM lesions facilitates sorcin recruitment and subsequent ESCRT-III assembly for PM repair.

### ANXA11 is sufficient to recruit sorcin to liposomes in the presence of calcium

**Figure 7.**
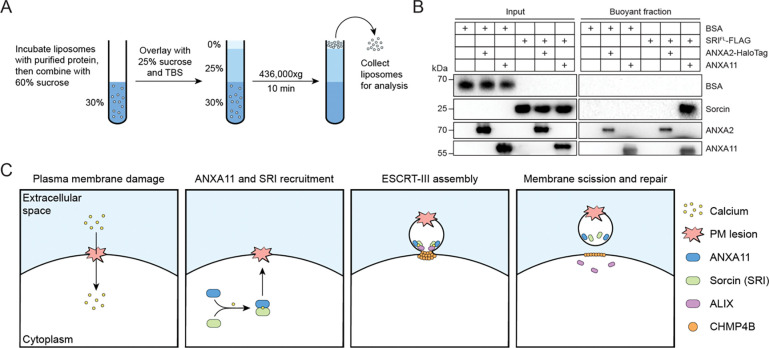
ANXA11 is sufficient to recruit sorcin to liposomes in the presence of calcium. (A) Schematic of the liposome flotation assay. Purified proteins were incubated with synthetic liposomes and calcium prior to flotation through a sucrose density gradient to determine which proteins associate with membranes. (B) Immunoblot analysis of the liposome flotation assays. The components of each reaction are indicated. (C) Schematic depicting the current working model of sorcin-mediated plasma membrane repair.

We directly tested a part of this model by performing liposome flotation assays with purified components (Fig. 7A). Recombinant sorcin was incubated with synthetic liposomes and calcium without or with recombinant annexin A2 (ANXA2) or ANXA11. The binding reactions were applied to the bottom of sucrose density gradients followed by high-speed centrifugation. Buoyant liposomes and bound proteins were solubilized then evaluated by immunoblot analysis. We found that sorcin alone was unable to float with liposomes, indicating that sorcin cannot directly bind membranes (Fig. 7B). However, sorcin floated with liposomes only when ANXA11, but not ANXA2, was added to the binding reaction. Bovine serum albumin (BSA) served as a negative control and did not float with liposomes under all tested conditions. These results indicated that sorcin binds directly and specifically to ANXA11, and that ANXA11 is sufficient to recruit sorcin to membranes in the presence of calcium (Fig. 7B).

## Discussion

Here, we identify sorcin as an early-acting membrane repair factor that facilitates ESCRT-III assembly at sites of PM damage (Fig. 7C). Our data support a model in which ANXA11, recruited to the PM upon damage-induced calcium influx, serves as an anchor that promotes the sequential recruitment of sorcin and ESCRT-III at PM lesions (Fig. 4–6). Sorcin directly and simultaneously interacts with ANXA11 and ALIX via its PEF domain and flexible N-terminus, respectively (Fig. 3D). Genetic depletion of sorcin sensitizes cells to PM damage inflicted by pore-forming toxin treatment (Fig. 2C–D) and impairs the recruitment of ESCRT-III, but not ANXA11, to PM lesions (Fig. 4E–F and 5).

ANXA11 depletion substantially decreases the size of the FM4–64-positive repair cap (Fig. 4C). We previously showed that ANXA2 depletion has a similar effect (Williams et al., 2025). Together, these data suggest that annexins have nonredundant roles in repair cap formation and that the proper balance of annexins is required for successful repair cap assembly. Additionally, sorcin binds to the proline-rich ANXA11 N-terminus, which is similar to the motif that we previously found is cleaved by calpains during PM repair. Thus, calpains may regulate ANXA11-mediated ESCRT-III recruitment.

The ANXA11/SRI pathway may synergize with the reported ANXA7/ALG-2 pathway to recruit ESCRT-III to PM lesions (Sønder et al., 2019). However, we have found some biochemical differences between sorcin and ALG-2. Our data indicate that sorcin simultaneously engages ANXA11 and ALIX via its flexible N-terminus and PEF domain, respectively. Additionally, sorcin is unable to directly bind membranes in the presence of calcium. In contrast, ALG-2 interacts with both ANXA7 and ALIX via its PEF domain (Tarabykina et al., 2000; Satoh et al., 2002) and has recently been reported to directly bind membranes (Shukla et al., 2022; Shukla et al., 2024). Thus, our data identify a mechanism in which annexin recruitment is directly coupled to ESCRT-III assembly during PM repair.

Genetic mutations in ESCRT machinery have been implicated in various neurodegenerative and membrane repair diseases (Skibinski et al., 2005; Zee et al., 2008; Rodger et al., 2020; Coyne et al., 2021; Coyne and Rothstein, 2021; Keeley and Coyne, 2024). In addition, ANXA11 mutations have been associated with ALS and muscular dystrophy (Smith et al., 2017; Johari et al., 2022; Martins et al., 2025). Although these mutations have been studied primarily in the context of lysosomal membrane repair, a recent report found that ALS- and FTD-associated mutations in ANXA11 and CHMP2B also compromise PM repair (Heffner et al., 2024). Our identification of sorcin as a factor that recruits ESCRT-III to PM lesions via ANXA11 raises the possibility that disease-associated ANXA11 mutations might impair PM repair, at least in part, by disrupting sorcin recruitment to PM lesions. Consistent with this suggestion, multiple disease-associated ANXA11 mutations map to the same flexible N-terminal region that binds sorcin.

Finally, our data highlight mechanistic and topological similarities between the budding of membrane-enveloped viruses (e.g. Human Immunodeficiency Virus [HIV-1]) and damage-induced PM vesicles (microvesicles). Göttlinger and colleagues provided the initial piece of evidence that host factors contribute to virus budding (Göttlinger et al., 1991). Subsequent work by the Sundquist and Göttlinger teams revealed that the p6 region of the HIV-1 Gag protein engages TSG101 and ALIX, which subsequently recruit ESCRT-III and Vps4 for membrane constriction and scission (Garrus et al., 2001; Strack et al., 2003; Fisher et al., 2007). More recently, we found that PM damage elicits extracellular vesicle secretion and that expression of a dominant-negative Vps4 mutant, which blocks ESCRT-III disassembly and vesicle scission, reduces the secretion of damage-induced microvesicles (Williams and Ngo et al., 2023; Williams et al., 2025). Our identification of sorcin as a membrane repair factor that localizes to PM lesions and recruits ESCRT-III for microvesicle shedding leads us to propose that enveloped viruses and damage-induced microvesicles share a common mechanism of membrane budding. Specifically, we propose that ANXA11 and sorcin function analogously to retroviral Gag proteins in recruiting ALIX and ESCRT-III to the PM for membrane budding. We speculate that membrane-enveloped viruses may have co-opted this host pathway of PM ESCRT recruitment to facilitate virion assembly and propagation.

## Materials and Methods

### Cell lines, media and general chemicals

HEK293T, HCT116 and U2-OS cell lines were cultured in 5% CO_2_ at 37°C and maintained in Dulbecco’s Modified Eagle Medium (DMEM) supplemented with 10% fetal bovine serum (FBS). Cells were routinely assessed, and found negative, for mycoplasma contamination using the MycoAlert Mycoplasma Detection Kit (Lonza Biosciences). All of the cell lines used in this study were authenticated by the UC Berkeley Cell Culture facility using STR profiling. Unless otherwise noted, all other chemicals were purchased from Sigma-Aldrich.

### Antibodies

Primary antibodies used for immunoblot (1:1000 dilution) were: mouse anti-ALIX (Santa Cruz sc-53540), mouse anti-BSA (Santa Cruz sc-32816), rabbit anti-annexin A1 (Abcam ab214486), rabbit anti-annexin A2 (Abcam ab185957), rabbit anti-annexin A11 (Proteintech 10479–2-AP), rabbit anti-HA-Tag (Cell Signaling Technology C29F4), rabbit anti-SRI (Abcam ab71983), and rabbit anti-vinculin (Abcam ab129002). Secondary antibodies used for immunoblotting (1:10,000 dilution) were: HRP-linked sheep anti-mouse IgG (Cytiva NA931) and HRP-linked donkey anti-rabbit IgG (Cytiva NA934).

### Plasmids

Lck-mEGFP-3xHA was cloned into a pLIX403-Puro backbone. mNeonGreen and mScarlet fusion proteins were cloned into pLJM1-Puro and pLJM1-Blast backbones, respectively. Constructs for recombinant protein purification were cloned into a pET28a backbone. Recombinant, full-length and truncated sorcin were appended at the C-terminus with a FLAG tag and a His_6_ tag. Recombinant ANXA2 was appended at the C-terminus with HaloTag and a His_6_ tag. All plasmid constructs were verified by whole-plasmid sequencing (ElimBio).

### Lentivirus production and transduction

HEK293T cells at ~40% confluence in a 6-well plate were transfected with 165 ng of pMD2.G, 1.35 μg of psPAX2 and 1.5 μg of a lentiviral plasmid using the TransIT-LT1 Transfection Reagent (Mirus Bio) according to the manufacturer’s protocol. The lentivirus-containing medium was harvested 48 h post-transfection by filtration through a 0.45 μm polyethersulfone filter (VWR Sciences). The filtered lentivirus was aliquoted, snap-frozen in liquid nitrogen and stored at −80°C. MDA-MB-231 cells were transduced with filtered lentivirus in the presence of 8 μg/ml polybrene for 24 h and then the medium was replaced. The cells were selected using 1 μg/ml puromycin or 5 μg/ml blasticidin.

### Immunoblotting

Cells were lysed in tris-buffered saline (TBS) supplemented with 1% TX-100 and a protease inhibitor cocktail (1 mM 4-aminobenzamidine dihydrochloride, 1 μg/ml antipain dihydrochloride, 1 μg/ml aprotinin, 1 μg/ml leupeptin, 1 μg/ml chymostatin, 1 mM phenylmethylsulfonyl fluoride (PMSF), 50 μM N-tosyl-L-phenylalanine chloromethyl ketone and 1 μg/ml pepstatin) and incubated on ice for 15 min. The whole cell lysate was centrifuged at 15,000×g for 10 min at 4°C, and the post-nuclear supernatant (PNS) was diluted with 6X Laemmli buffer (reducing) to a 1X final concentration. Samples were heated at 95°C for 5 min and resolved in 4–20% acrylamide Tris-glycine gradient gels (Life Technologies). The resolved proteins were then transferred to polyvinylidene difluoride (PVDF) membranes (EMD Millipore).

For immunoblots, the PVDF membranes were blocked for 30 min with 5% bovine serum albumin (BSA) in TBS supplemented with 0.1% Tween-20 (TBS-T) and incubated overnight with 1:1000 dilutions of primary antibodies in 5% BSA in TBS-T. The membranes were then washed three times with TBS-T, incubated for 1 h at room temperature with 1:10,000 dilutions of HRP-conjugated secondary antibodies (Cytiva Life Sciences) in 5% BSA in TBS-T, washed three times with TBS-T, washed once with tris-buffered saline (TBS) and then detected with ECL2 or PicoPLUS reagent (Thermo Fisher Scientific).

### Inside-out plasma membrane vesicle immunopurification and mass spectrometry

HCT116 Lck-mEGFP-3xHA cells were grown to ~90% confluence in 6 × 150 mm dishes. Lck-mEGFP-3xHA expression was induced by replacing the conditioned medium with DMEM supplemented with 10% FBS and 0.125 μg/ml doxycycline 24 h prior to the experiment. All subsequent manipulations were performed at 4°C. Each 150 mm dish was washed once with 5 ml of cold PBS supplemented with 1 mM EGTA then harvested by scraping into 5 ml of cold PBS containing 1 mM EGTA. The cells were collected by centrifugation at 300×g for 5 min, and the supernatant was discarded. The cell pellets were resuspended in 2 vol of organelle lysis buffer (1X TBS, 1 mM EGTA, 5 mM TCEP, 25 μM Calpain-I inhibitor and a protease inhibitor cocktail (see immunoblotting section)). The cell suspension was then mechanically lysed by passing through a 25-gauge needle until ~85% of cells were lysed as assessed by trypan blue exclusion. The lysed cells were centrifuged at 1,000×g for 15 min to sediment intact cells and nuclei, and the PNS was transferred to a 15 ml conical tube.

Beads from 200 μl of a magnetic Protein G Dynabead (Thermo Fisher Scientific) slurry were sedimented with a magnetic tube rack and resuspended in organelle lysis buffer. The bead slurry was split evenly into four tubes and re-sedimented. The PNS was equally divided and dispensed between the four tubes. Tubes #1 and #2 received 5 μg of mouse IgG isotype antibody (Cell Signaling Technology G3A1), and tubes #3 and #4 received 5 μg of mouse anti-HA antibody (Proteintech 66006–2-Ig). Tubes #2 and #4 also received CaCl_2_ (2 mM final). The binding reactions were incubated on a rotating mixer for 15 min and washed three times with organelle lysis buffer. The wash buffer for tubes #2 and #4 was supplemented with 2 mM CaCl_2_. Each sample was eluted by heating at 55°C for 5 min in 65 μl of elution buffer (50 mM TEAB and 0.1% Rapigest (Waters)). An aliquot of each eluate (15 μl) was reserved for Sypro Ruby staining and immunoblot analysis. The remaining eluates from tubes #3 and #4 (three biological replicates each) were sent to the UC Berkeley Vincent J. Coates proteomics facility for label-free quantitative mass spectrometry according to their standards. The mass spectrometry data were analyzed using PEAKS Studio.

### Microscopy and laser ablation

Images were acquired using an LSM900 confocal microscope system (ZEISS) using confocal mode, a 63× Plan-Apochromat, NA 1.40 objective, and a heated, CO_2_ controlled chamber. Cells were imaged in Fluorobrite DMEM supplemented with 2.5 μM FM4–64 (Biotium). For laser ablation experiments, a 1 μm × 1 μm square was positioned over the edge of a cell not adjacent to another cell. The 1 μm × 1 μm square was ablated for 100 iterations using all lasers at 100% power. Protein recruitment was quantified in Zen 3.1 (Zeiss) by measuring fluorescence intensities within a box that encapsulated the repair cap. Fluorescence intensities of an adjacent, non-ablated area of the membrane was measured and used for a frame-by-frame background subtraction. The recruitment graphs plot the background-corrected change in fluorescence intensity divided by the fluorescent intensity at the damage site prior to ablation.

For the laser ablation experiments in Figure 2B, the cells were washed once with TBS and imaged in TBS supplemented with 2.5 μM FM4–64 and either 2 mM CaCl_2_ or 2 mM EGTA.

### RNA interference

U2-OS cells were suspended by trypsinization and transfected using Lipofectamine RNAiMAX (Thermo Fisher Scientific) according to the manufacturer’s instructions. The cell suspension was diluted in conditioned medium to a final concentration of 1.5 × 10^5^ cells/ml, 20 nM total siRNA, and 0.3% v/v transfection reagent, dispensed into individual wells of a poly-D-lysine-coated six-well plate and incubated overnight at 37°C. Conditioned medium was replaced after 18–24 h. At 48 h post-transfection, cells were suspended by trypsinization, reseeded at an appropriate density for laser ablation or cell viability experiments and incubated at 37°C for an additional 24 h. The following siRNAs were purchased from Qiagen: ANXA11 (Hs_ANXA11_6), SRI (Hs_SRI_10) and a non-targeting control (1027310).

### Cell viability assays

U2-OS cells were transfected with siRNAs as above, seeded in a poly-D-lysine-coated 24-well plate and grown to 70% confluence. At 72 h post-transfection, cells were treated with vehicle or 25 ng/ml PFO for 30 min in 200 μl of Fluorobrite DMEM. Fluorobrite DMEM was replaced with 200 μl of fresh DMEM + 10% FBS, and the cells were allowed to recover for 1 h at 37°C. After the recovery period, 200 μl of Fluorobrite DMEM supplemented with 0.5 mg/ml MTT reagent was added to each well and incubated for 1 h at 37°C. The MTT reagent was removed, replaced with 200 μl of DMSO, and incubated for 5 min. An aliquot of each DMSO suspension (100 μl) was transferred to a 96-well plate, and the absorbance was measured at 570 nm. To determine cell viability, we subtracted the background from a DMSO blank from each sample measurement.

### Recombinant protein purification

Recombinant sorcin and ANXA2 proteins were expressed in *E. coli* Rosetta2(DE3)pLysS cells. Pre-cultures (30 ml) were grown overnight at 37°C and diluted to 3000 ml cultures. The cultures were incubated at 37°C until the OD_600_ reached ~0.6, and protein expression was induced upon addition of 0.2 mM IPTG for 4 h at 37°C. The cells were harvested by centrifugation at 5,000×g for 10 min in a swing-bucket H-6000A rotor, and cell pellets were stored at −80°C until use. All subsequent manipulations were performed at 4°C. The cell pellets were resuspended in 30 ml of cold Ni-NTA lysis buffer (20 mM Tris-HCl pH 8.0, 300 mM NaCl, 10 mM imidazole and a protease inhibitor cocktail) and sonicated 5 times (5 sec on, 15 sec off, 20% power). Each lysate was clarified by centrifugation at 20,000×g for 15 min in a fixed angle FIBERlite F21–8x50y rotor, and the supernatant fractions were applied to gravity flow columns containing 1 ml of pre-equilibrated HisPur^™^ Ni-NTA Resin (Thermo Fisher Scientific). Each column was washed with three column volumes of cold Ni-NTA wash buffer (similar recipe as Ni-NTA lysis buffer but with 25 mM imidazole) and eluted with cold Ni-NTA elution buffer (similar recipe as Ni-NTA lysis buffer but with 300 mM imidazole). Recombinant perfringolysin O (PFO) was purified as in Williams and Ngo et al., 2026. Recombinant His_6_-ANXA11 was purchased from Bio-Techne.

### Sorcin immunoprecipitation

HCT116 wild-type cells were grown to ~90% confluence in 10 × 150 mm dishes. All subsequent manipulations were performed at 4°C unless otherwise indicated. Each 150 mm dish was washed once with 5 ml of cold PBS supplemented with 1 mM EGTA then harvested by scraping into 5 ml of cold PBS containing 1 mM EGTA. Cells were pooled, collected by centrifugation at 300×g for 5 min, and the supernatant was discarded. The cell pellet was then resuspended in 2 vol of organelle lysis buffer, and the cell suspension was mechanically lysed by passing through a 25-gauge needle until ~85% of cells were lysed as assessed by trypan blue exclusion. The lysed cells were centrifuged at 1,000×g for 15 min to sediment intact cells and nuclei, and the PNS was then centrifuged at 100,000×g for 30 min in an SW55 rotor. The supernatant (cytosol fraction) was collected conservatively without disturbing the membrane pellet. The purified cytosol was diluted 1:2 in organelle lysis buffer.

In parallel, 150 μl of anti-FLAG agarose beads (Chromotek ffa-20) were sedimented at 1,000×g for 2 min and resuspended in 600 μl of organelle lysis buffer. The resuspended beads were split evenly into six tubes. FLAG (3x) peptide, SRI^FL^-FLAG and SRI^Δ1–32^-FLAG baits (4 μM each) were added to anti-FLAG beads in a 200 μl reaction. Bait proteins were bound to the anti-FLAG beads for 1 h at 4°C. The beads were then washed three times with organelle lysis buffer and sedimented at 1,000×g for 2 min. The purified cytosol was equally divided and dispensed between the six tubes. CaCl_2_ (2 mM final) was added to the indicated tubes. The binding reactions were incubated on a rotating mixer for 2 h. Beads were then washed three times with organelle lysis buffer. The wash buffer for the calcium immunoprecipitations was supplemented with 2 mM CaCl_2_. Each sample was eluted in 1X Laemmli buffer (non-reducing) for immunoblot analysis.

### Liposome production

To generate liposomes, lipids were mixed in a glass vial in the following molar ratios: 38:20:20:3:0.5:18.5 DOPC:POPE:DOPS:PI(4,5)P_2_:TexasRed-PE:cholesterol (1 μmol total lipid) in 500 μl of 5% methanol and 95% chloroform. The lipid mixture was placed on a heat block preheated to 55°C, and the organic solvent was evaporated under vacuum for 30 min. Dried lipids were resuspended in 500 μl of degassed TBS and incubated at 55 °C with intermittent vortexing for 15 min. The lipid solution was pumped 11 times through an extruder with a 200-nm filter.

### Buoyant density gradient flotation

For sucrose density gradient flotation assays, binding reactions (60 μl) consisted of liposomes (20 μl), purified proteins, TBS and CaCl_2_ (2 mM final). The reactions were incubated at room temperature for 15 min, mixed with 60 μl of 60% sucrose in TBS + 2 mM CaCl_2_ (for a final concentration of 30% sucrose), and sequentially overlaid with 100 μl of 25% sucrose in TBS + 2 mM CaCl_2_ and 20 μl of TBS + 2 mM CaCl_2_. The resulting sucrose step gradients were centrifuged in a TLA100 rotor (Beckman) at 100,000 rpm (436,000×g) for 10 min at 4°C. Buoyant liposomes were collected, and the bound proteins were analyzed by SDS-PAGE and immunoblotting.

### Statistical analysis

Statistical analyses were performed using GraphPad Prism 10. Unpaired two-tailed Student’s *t-*tests were used for comparisons of two groups, and one- or two-way analysis of variance (ANOVA) tests were used for group comparisons. The statistical test used is detailed in each figure legend.

## Supplementary Material

Supplement 1 **Movie 1. Laser ablation of U2-OS SRI-mNG cells.** U2-OS cells stably expressing SRI-mNG were subjected to laser ablation. Green: SRI-mNG; Magenta: FM4–64. Image times are relative to the first image taken after laser ablation. Total imaging time: 5 min. Time between acquisitions: 3 s. Scale bars: 5 μm. See corresponding Figure 2A.

Supplement 2 **Movie 2. Laser ablation of U2-OS SRI-mNG cells in the presence of extracellular calcium.** U2-OS cells stably expressing SRI-mNG were subjected to laser ablation in the presence of extracellular calcium. Green: SRI-mNG; Magenta: FM4–64. Image times are relative to the first image taken after laser ablation. Total imaging time: 5 min. Time between acquisitions: 3 s. Scale bars: 5 μm. See corresponding Figure 2B.

Supplement 3 **Movie 3. Laser ablation of U2-OS SRI-mNG cells in the absence of extracellular calcium.** U2-OS cells stably expressing SRI-mNG were subjected to laser ablation in the absence of extracellular calcium. Green: SRI-mNG; Magenta: FM4–64. Image times are relative to the first image taken after laser ablation. Total imaging time: 5 min. Time between acquisitions: 3 s. Scale bars: 5 μm. See corresponding Figure 2B.

Supplement 4 **Movie 4. Laser ablation of U2-OS SRI-mNG siNT cells.** U2-OS cells stably expressing SRI-mNG were transfected with a non-targeting siRNA (siNT) and subjected to laser ablation. Green: SRI-mNG; Magenta: FM4–64. Image times are relative to the first image taken after laser ablation. Total imaging time: 5 min. Time between acquisitions: 3 s. Scale bars: 5 μm. See corresponding Figure 4C.

Supplement 5 **Movie 5. Laser ablation of U2-OS SRI-mNG siANXA11 cells.** U2-OS cells stably expressing SRI-mNG were transfected with an ANXA11-targeting siRNA (siANXA11) and subjected to laser ablation. Green: SRI-mNG; Magenta: FM4–64. Image times are relative to the first image taken after laser ablation. Total imaging time: 5 min. Time between acquisitions: 3 s. Scale bars: 5 μm. See corresponding Figure 4C.

Supplement 6 **Movie 6. Laser ablation of U2-OS ANXA11-mSc siNT cells.** U2-OS cells stably expressing ANXA11-mSc were transfected with a non-targeting siRNA (siNT) and subjected to laser ablation. Green: ANXA11-mSc; Magenta: FM4–64. Image times are relative to the first image taken after laser ablation. Total imaging time: 5 min. Time between acquisitions: 3 s. Scale bars: 5 μm. See corresponding Figure 4E.

Supplement 7 **Movie 7. Laser ablation of U2-OS ANXA11-mSc siSRI cells.** U2-OS cells stably expressing ANXA11-mSc were transfected with a sorcin-targeting siRNA (siSRI) and subjected to laser ablation. Green: ANXA11-mSc; Magenta: FM4–64. Image times are relative to the first image taken after laser ablation. Total imaging time: 5 min. Time between acquisitions: 3 s. Scale bars: 5 μm. See corresponding Figure 4E.

Supplement 8 **Movie 8. Laser ablation of U2-OS ALIX-mNG siNT cells.** U2-OS cells stably expressing ALIX-mNG were transfected with a non-targeting siRNA (siNT) and subjected to laser ablation. Green: ALIX-mNG; Magenta: FM4–64. Image times are relative to the first image taken after laser ablation. Total imaging time: 5 min. Time between acquisitions: 3 s. Scale bars: 5 μm. See corresponding Figure 5A.

Supplement 9 **Movie 9. Laser ablation of U2-OS ALIX-mNG siSRI cells.** U2-OS cells stably expressing ALIX-mNG were transfected with a sorcin-targeting siRNA (siSRI) and subjected to laser ablation. Green: ALIX-mNG; Magenta: FM4–64. Image times are relative to the first image taken after laser ablation. Total imaging time: 5 min. Time between acquisitions: 3 s. Scale bars: 5 μm. See corresponding Figure 5A.

Supplement 10 **Movie 10. Laser ablation of U2-OS ALIX-mNG siANXA11 cells.** U2-OS cells stably expressing ALIX-mNG were transfected with an ANXA11-targeting siRNA (siANXA11) and subjected to laser ablation. Green: ALIX-mNG; Magenta: FM4–64. Image times are relative to the first image taken after laser ablation. Total imaging time: 5 min. Time between acquisitions: 3 s. Scale bars: 5 μm. See corresponding Figure 5A.

Supplement 11 **Movie 11. Laser ablation of U2-OS CHMP4B-mNG siNT cells.** U2-OS cells stably expressing CHMP4B-mNG were transfected with a non-targeting siRNA (siNT) and subjected to laser ablation. Green: CHMP4B-mNG; Magenta: FM4–64. Image times are relative to the first image taken after laser ablation. Total imaging time: 5 min. Time between acquisitions: 3 s. Scale bars: 5 μm. See corresponding Figure 5C.

Supplement 12 **Movie 12. Laser ablation of U2-OS CHMP4B-mNG siSRI cells.** U2-OS cells stably expressing CHMP4B-mNG were transfected with a sorcin-targeting siRNA (siSRI) and subjected to laser ablation. Green: CHMP4B-mNG; Magenta: FM4–64. Image times are relative to the first image taken after laser ablation. Total imaging time: 5 min. Time between acquisitions: 3 s. Scale bars: 5 μm. See corresponding Figure 5C.

Supplement 13 **Movie 13. Laser ablation of U2-OS CHMP4B-mNG siANXA11 cells.** U2-OS cells stably expressing CHMP4B-mNG were transfected with an ANXA11-targeting siRNA (siANXA11) and subjected to laser ablation. Green: CHMP4B-mNG; Magenta: FM4–64. Image times are relative to the first image taken after laser ablation. Total imaging time: 5 min. Time between acquisitions: 3 s. Scale bars: 5 μm. See corresponding Figure 5C.
